# Scanning Deposition Method for Large-Area Diamond Film Synthesis Using Multiple Microwave Plasma Sources

**DOI:** 10.3390/nano12121959

**Published:** 2022-06-08

**Authors:** Seung Pyo Hong, Kang-il Lee, Hyun Jong You, Soo Ouk Jang, Young Sup Choi

**Affiliations:** Institute of Plasma Technology, Korea Institute of Fusion Energy, 37 Dongjangsan-ro, Gunsan-si 54004, Jeollabuk-do, Korea; sphong@kfe.re.kr (S.P.H.); kilee@kfe.re.kr (K.-i.L.); hjyou@kfe.re.kr (H.J.Y.); sojang@kfe.re.kr (S.O.J.)

**Keywords:** diamond synthesis, microwave plasma, large-area, surface-wave plasma, diamond film, scanning deposition

## Abstract

The demand for synthetic diamonds and research on their use in next-generation semiconductor devices have recently increased. Microwave plasma chemical vapor deposition (MPCVD) is considered one of the most promising techniques for the mass production of large-sized and high-quality single-, micro- and nanocrystalline diamond films. Although the low-pressure resonant cavity MPCVD method can synthesize high-quality diamonds, improvements are needed in terms of the resulting area. In this study, a large-area diamond synthesis method was developed by arranging several point plasma sources capable of processing a small area and scanning a wafer. A unit combination of three plasma sources afforded a diamond film thickness uniformity of ±6.25% at a wafer width of 70 mm with a power of 700 W for each plasma source. Even distribution of the diamond grains in a size range of 0.1–1 μm on the thin-film surface was verified using field-emission scanning electron microscopy. Therefore, the proposed novel diamond synthesis method can be theoretically expanded to achieve large-area films.

## 1. Introduction

Diamonds are known as a material with excellent electrical, thermal, mechanical, and optical properties, which comprise a tetrahedral structure with strong sp^3^ covalent bonds. Therefore, diamonds are widely used not only for their value as jewelry but also in the fields of mechanical structures, processing tools, and semiconductor devices. In addition, research on their application in various fields, such as micro-electro-mechanical systems (MEMS), optics, electronic-emission devices, and sensors, is being actively conducted [1,2,3,4,5,6,7]. In particular, diamonds are being evaluated as an ideal next-generation material in semiconductor devices. They are also considered a cutting-edge material suitable for quantum computers and space electronic devices in radiation environments, owing to their high electron and hole mobility and their excellent bandgap energy characteristics [8]. The recent increase in frequency bands with the development of wireless communication technology to 5G and beyond has prompted the need for high-performance power semiconductor devices. Further, this entails improvement in the heat dissipation efficiency of the device, thereby increasing the demand for diamond substrates [9].

Since General Electric reported artificial diamond synthesis using a high-pressure and high-temperature method in 1955, different diamond deposition techniques have been extensively investigated [10]. Diamond film growth from the vapor phase on a nondiamond substrate has been accomplished by developing thermal- and plasma-enhanced chemical vapor deposition (CVD) methods. Various sophisticated techniques have been developed for diamond applications, including filament-assisted thermal CVD [11,12], electron-assisted thermal CVD [13,14], laser-assisted thermal CVD [15], RF-plasma CVD [16], microwave plasma CVD [17], combustion flame-assisted CVD [18], and direct-current arc plasma jet CVD [19].

Among the various techniques for diamond film deposition, microwave plasma chemical vapor deposition (MPCVD) has been widely used in diverse applications. For low-pressure resonant cavity MPCVD, a high gas temperature is maintained by concentrating microwaves near the substrate, which allows the rapid growth of a high-quality diamond thin film [20]. However, this method is difficult to use for a large-area substrate, such as 150 mm or larger, because it is not easy to control plasma distribution uniformly due to the short wavelength of microwaves.

Surface-wave plasma CVD using microwaves has been suggested as an alternative to solve the abovementioned problem since it is comparatively easier to form a large-area film [21]. The development of an advanced CVD method for large-area diamond synthesis has, however, been challenging [22,23]. Although considerable research on the synthesis of large-area diamonds has been conducted, diamond crystallinity and deposition rate still require improvement. In this study, we developed a new method to produce a large-area diamond film using surface-wave plasma with increasing diamond crystallinity. Several surface-wave plasma generators were installed in the upper section of a vacuum chamber, and a constant CH_4_/H_2_ gas ratio was injected into the chamber to synthesize diamond films on an Si wafer at pressures of 100 mTorr or higher.

## 2. Materials and Methods

**Figure 1:**
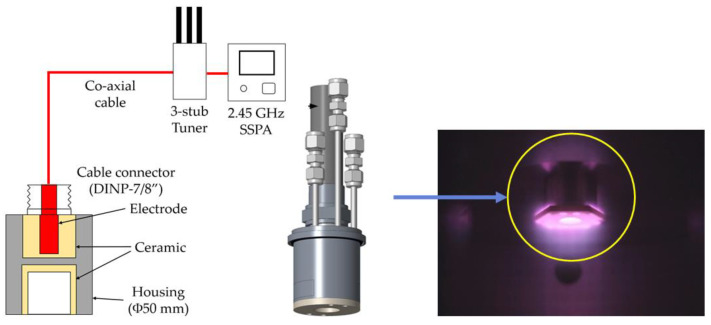
Schematic of the microwave single-launcher plasma source and optical image of the plasma discharge during the deposition process.

Surface-wave plasma is easily generated at low pressures (<1 torr). Although improved results have been reported when employing surface-wave plasma at low pressures [24], nanodiamonds with low crystallinity are produced because of low gas temperatures compared to established microwave techniques that operate at 100–300 Torr. Therefore, the gas temperature should be enhanced by concentrating plasma in a specific region to synthesize high-purity microcrystalline diamonds (MCDs) in the aforementioned environment. In a previous study, individual surface-wave plasma generators with excellent scalability were constructed to validate the performance of the remote plasma process [25]. In this study, the plasma source from the previous study was improved to facilitate diamond film deposition, as shown in Figure 1.

The improved structure comprised a microwave coupling space and a plasma generation region. The length of the source region in which the plasma was generated was shortened to extract the plasma in the shape of a ball; furthermore, the microwave transmission part was replaced with a coaxial cable from a rectangular waveguide to allow the mounting of several surface-wave plasma sources. Ball-shaped plasma was generated by concentrating the microwaves in the inner space of the plasma source (inner diameter 20 mm × depth 20 mm) using a 2.45 GHz solid-state power amplifier and a three-stub tuner.

**Figure 2:**
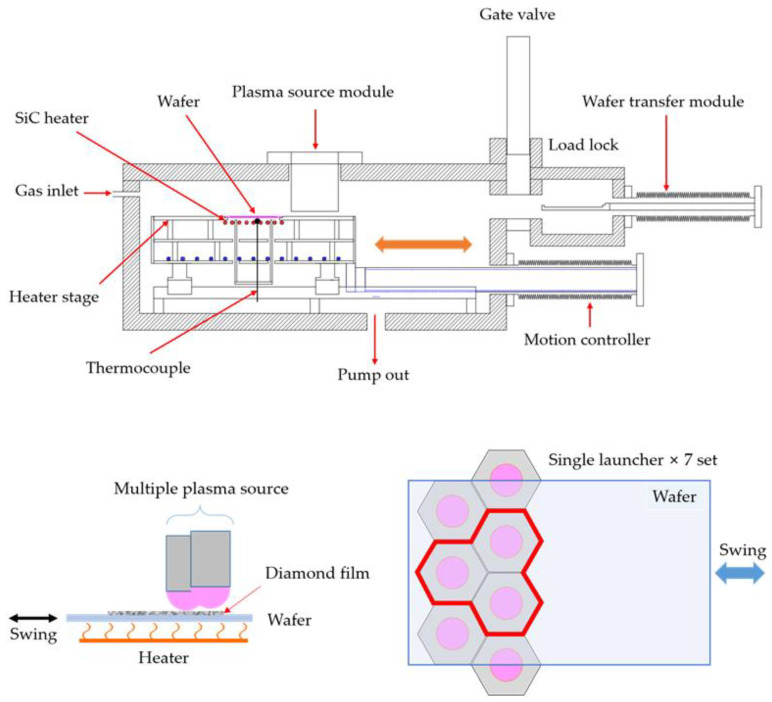
Schematic of the scanning deposition system and multiple microwave plasma source array.

As shown in Figure 2, a microwave single-launcher source array was mounted in the upper part of the process chamber, and a SiC heater was installed in the process chamber to heat the substrate to a maximum temperature of 1000 °C. Substrate with a maximum size of 150 mm was placed on the upper stage of the SiC heater using a load lock, and its temperature was controlled using a thermocouple (TC) in close contact with the backside of the substrate. Process gases, including Ar, H_2_, and CH_4_, were supplied to the chamber through the mass flow controllers. The substrate was located 10–20 mm below the plasma source and was in direct contact with the ball-shaped plasma. It was difficult to deposit the diamond thin film uniformly over a large area in a structure where the source and substrate were fixed when the effective deposition area of each source was smaller than the outer diameter of the plasma source. In particular, the film thickness had a nonuniform distribution with the maximum thickness near the center of the source. Multiple sources were arranged linearly in a two-row, zigzag pattern to reduce the space between each source. A diamond film was uniformly deposited by reciprocating the substrate perpendicular to the source arrangement direction. The main process conditions are listed in Table 1.

The surface morphologies and thicknesses of the grown films were investigated using field-emission scanning electron microscopy (FE-SEM) (Sigma, Carl Zeiss, Jena, Germany). The crystal orientation and the average crystallite size were determined using X-ray diffraction (Empyrean, Malvern Panalytical, Malvern, UK) with Cu Kα radiation (λ = 0.154 nm, 40 kV, 30 mA). To distinguish between diamond, graphite, and amorphous carbon in a diamond thin film, UV-Raman spectroscopy (InVia Raman Microscope, Renishaw, Wotton-under-Edge, UK) was performed in a back-scattering geometry with an excitation frequency of 325 nm (He-Cd laser). The integration time and the objective magnification were set to 180 s and 100×, respectively. The laser power was maintained within a range of 10%. Fixed-point subtraction was used for background subtraction in the range of 900–2000 cm^−1^. The spatial resolution of the UV-Raman spectroscopy was 0.3 cm^−1^. The sp^3^/sp^2^ carbon ratio of the diamond film was evaluated as the intensity ratio (I_Dia_/I_G_) of the designated diamond peak at ~1332 cm^−1^ (I_Dia_) to that of the G band at 1590 cm^−1^ (I_G_) after background subtraction.

## 3. Results and Discussion

### 3.1. Process Improvement for Diamond Synthesis Using the Single Plasma Source

**Figure 3:**
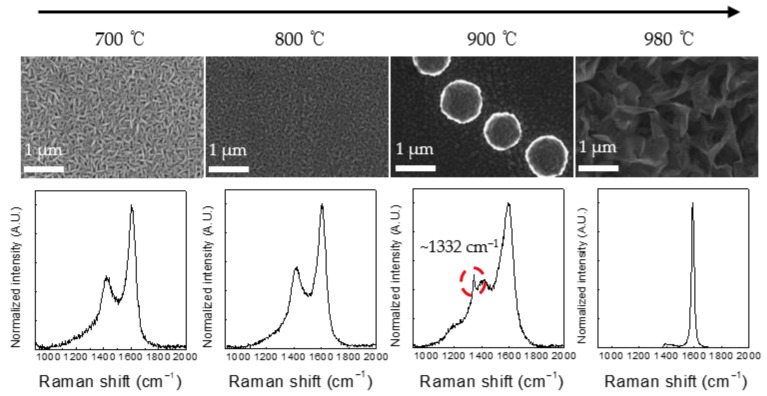
SEM images of the sample surface (**top**) and UV-Raman spectra of the samples (**bottom**) at various temperatures.

Process pressure, gas composition ratio, substrate temperature, and substrate surface treatment are significant variables that impact diamond film quality and the deposition rate in the diamond synthesis process employing H_2_ and CH_4_ gas-based plasma. The features of the synthetic diamond film produced using a single surface-wave plasma source were studied by performing several tests on the aforementioned primary variables. Figure 3 (top) shows the FE-SEM images of the film surface prepared using plasma with a gas mixture (CH_4_/H_2_ ratio of 1.0%) of 30 sccm Ar, 3 sccm CH_4_, and 300 sccm H_2_ at a microwave power of 800 W, an operating pressure of 700 mTorr, and different substrate temperatures. Silicon (100) wafers scratched with a 3 μm diamond abrasive were used as the substrate.

A thin film with distinct properties was created after performing the film deposition process for 3 h at substrate temperatures of 700, 800, 900, and 980 °C. Elongated amorphous carbon structures with sizes in the range of approximately 100–300 nm were produced at substrate temperatures of 700 and 800 °C. More dense amorphous carbons were formed under elevated temperatures. At a substrate temperature of 900 °C, diamonds with a size of approximately 1 µm were formed in a spherical shape within a matrix of amorphous carbons. Spherical-shaped diamonds tended to form along the directions of the fine scratches, which favored diamond nucleation, rather than on the entire substrate, resulting in a low number of density. At a substrate temperature of 980 °C, vertically oriented graphene (graphene nanowall) of several μm thickness was produced on the entire surface of the substrate.

Figure 3 (bottom) shows the UV-Raman analysis results of the thin films deposited under different conditions. The peak at ~1332 cm^−1^ corresponded to a diamond, and its breadth could be attributed to the small size of the diamond grains. The UV-Raman peak at ~1400 cm^−1^ indicated the disorder-induced double-resonance D band in graphitic carbon, and the broad peak at ~1590 cm^−1^ was attributed to sp^2^-bonded carbon [26,27,28,29,30,31]. Typical characteristic curves of amorphous carbon were obtained at 700 and 800 °C, whereas graphite characteristics were observed at 980 °C. At a substrate temperature of 900 °C, a weak diamond peak was observed at ~1332 cm^−1^, indicating low crystallinity.

**Figure 4:**
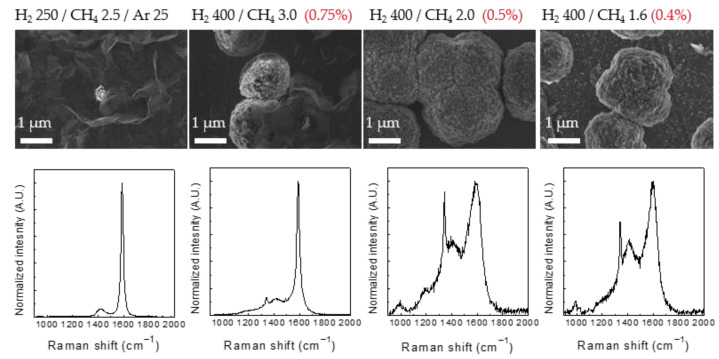
SEM images of the surface of samples at various CH_4_/H_2_ composition ratios, and UV-Raman spectra of each sample.

Figure 4 shows the experimental results with different CH_4_-to-H_2_ composition ratios. Diamond synthesis experiments were performed with CH_4_/H_2_ ratios of 1.00%, 0.75%, 0.50%, and 0.40% at a microwave-applied power of 800 W, a substrate temperature of 950 °C, and a process time of 6 h. A graphene nanowall was synthesized with a CH_4_/H_2_ ratio of 1.00%. Meanwhile, a graphene nanowall and nanodiamond were simultaneously synthesized at a CH_4_/H_2_ ratio of 0.75%. Considering that the graphene nanowall did not form when the CH_4_/H_2_ ratio was 0.50%, the composition ratio of 0.75% was assumed to be the transition region between the graphene nanowall and the nanodiamond. The UV-Raman measurements at the CH_4_/H_2_ ratios of 0.50% and 0.40% revealed clear diamond peaks at ~1332 cm^−1^.

**Figure 5:**
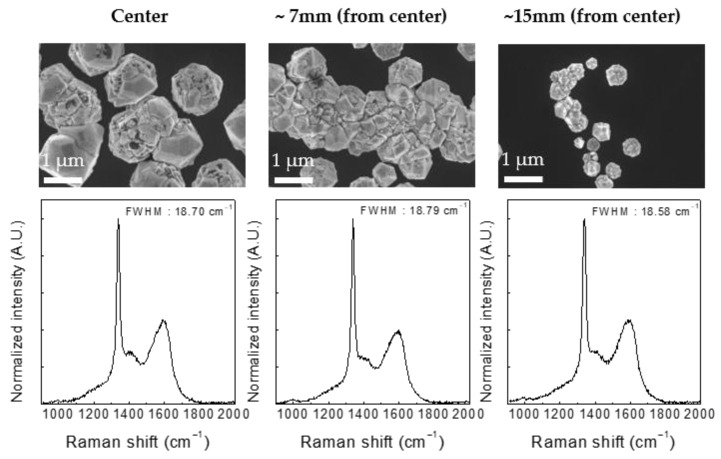
SEM images and UV Raman spectra of the samples deposited under a microwave power of 800 W, a pressure of 600 mTorr, and a temperature of 950 °C with a CH_4_/H_2_ gas mixture (1.5 sccm CH_4_ and 300 sccm H_2_).

Figure 5 shows the results of using the improved plasma and process conditions. Diamond film deposition was conducted with a CH_4_/H_2_ gas mixture comprising 1.5 sccm CH_4_ and 300 sccm H_2_ at a microwave power of 800 W, a pressure of 600 mTorr, and a substrate temperature of 950 °C. MCDs up to 1 μm in size were observed with improved crystallinity. The synthetic area was 30 mm in diameter, and the diamonds were irregularly distributed along the fine scratches. The UV-Raman spectrum revealed a diamond peak at 1338 cm^−1^. The shift of the diamond peak to 1338 cm^−1^ was attributed to the existence of several Raman lines and residual compressive stress [32,33]. The full width at half maximum (FWHM) of the Raman peak near 1332 cm^−1^ for each sample was 18.70, 18.79, and 18.58 cm^−1^, respectively. From the UV-Raman measurements, the I_Dia_/I_G_ ratio, which is directly associated with the sp^3^/sp^2^ carbon ratio, reached 2.75. The particle size significantly decreased with the increase in the distance from the center of the plasma; however, the I_Dia_/I_G_ ratio, which indicates the purity of the diamond, remained constant. The quality of the diamond thin film produced using the single plasma source was comparable to that of previous studies using surface-wave plasma at low pressures [34]. Furthermore, a diamond film with a grain size of 1 μm or more could be synthesized, and its quality could be further improved by adding CO_2_ or with post-treatment [24].

**Figure 6:**
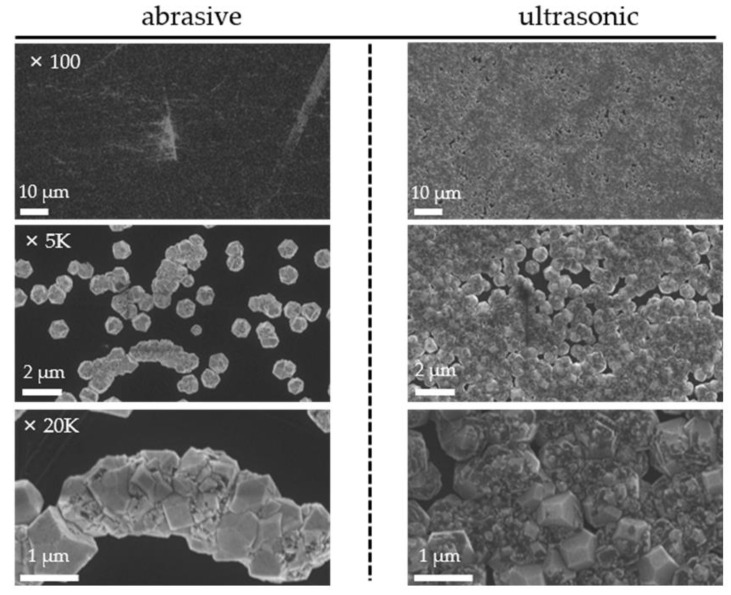
SEM images of the substrate surface after 6 h of deposition with substrates prepared via mechanical scratching and ultrasonic seeding methods at various magnifications.

As diamond synthesis is based on nucleation, the deposition rate can be enhanced by increasing the nucleation density of the substrate surface. The direct mechanical scratching of the substrates using 3 μm abrasives can promote nucleation by forming a strong electric field around the scratches, thereby enhancing crystallinity. However, this results in a low nucleation density because the nucleation of the diamond crystals is primarily observed on the defects of the mechanically damaged wafers and is clustered together around them [35,36]. Therefore, this scratching method is rarely used in large-area diamond synthesis. An ultrasonic seeding method is currently the most widely utilized approach in diamond synthesis [37]. In this experiment, a 5% solution was prepared using nanodiamond powder with a core particle size of 3–10 nm and ethanol. The surface of the silicon wafer was immersed in the colloidal solution and ultrasonicated for 5 min to manufacture a seed substrate. The SEM images of the substrates synthesized for 6 h using the mechanical scratching and ultrasonic methods are shown in Figure 6. For the substrate treated with the ultrasonic method, the film density was approximately 2.5 times higher than that treated with the scratch method. Moreover, the nucleation density of the substrates produced using the ultrasonic method could potentially be improved by optimizing the process conditions, such as the core particle size, concentration, and treatment time.

**Figure 7:**
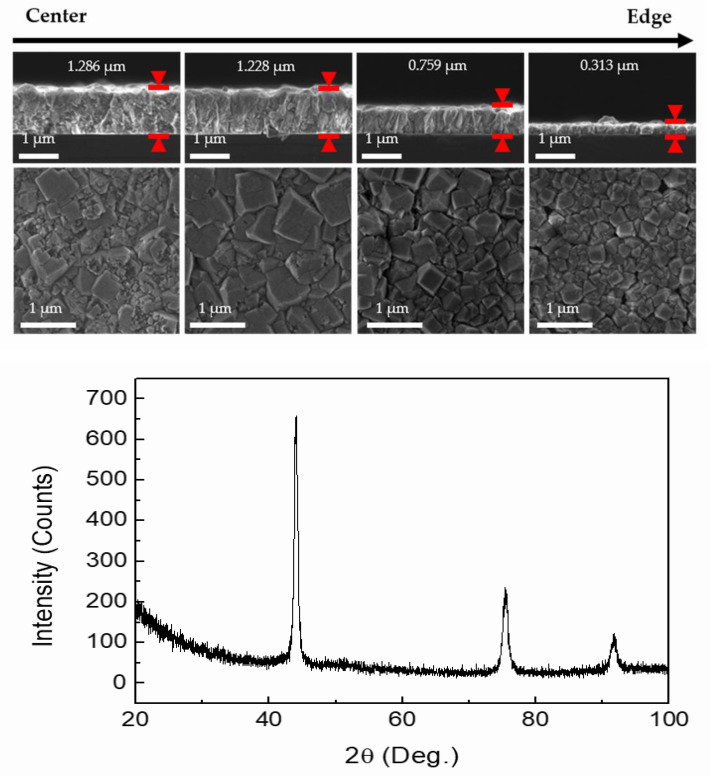
SEM images of the cross-sections and plan-views of the samples after deposition for 12 h (**top**), and the X-ray diffraction pattern of the deposited diamond film using Cu Kα radiation (**bottom**).

The process conditions for the microwave single plasma source in the newly designed diamond CVD were improved through a set of experiments. The film growth experiments were conducted at a microwave power of 800 W, a pressure of 600 mTorr, and a temperature of 950 °C with a CH_4_/H_2_ gas mixture (1.5 sccm CH_4_ and 300 sccm H_2_) for 12 h to observe the diamond film distribution using a single plasma source, as shown in Figure 7 (top). The thickness and surface morphology of the diamond film were measured according to the substrate position. The diamond film was deposited on an area with a diamond of 50 mm. The center and outer thicknesses were observed to be approximately 1.3 and 0.3 μm, respectively. The deposition rate tended to decrease significantly as the distance from the center increased. The deposited diamond film mainly grew with a cubic shape. Similar to the film thickness, the grain size tended to decrease as the measurement was obtained farther from the center. The XRD pattern for the diamond film revealed diffraction peaks at 44.0°, 75.4°, and 91.5°, which are typical of diamond crystalline planes (111), (220), and (311), as shown in Figure 7 (bottom). The average crystallite size was calculated as 14.9 nm from the FWHM of the (111) diamond diffraction peak.

### 3.2. Large-Area Diamond Synthesis Using a Multi-Surface-Wave Plasma Source

**Figure 8:**
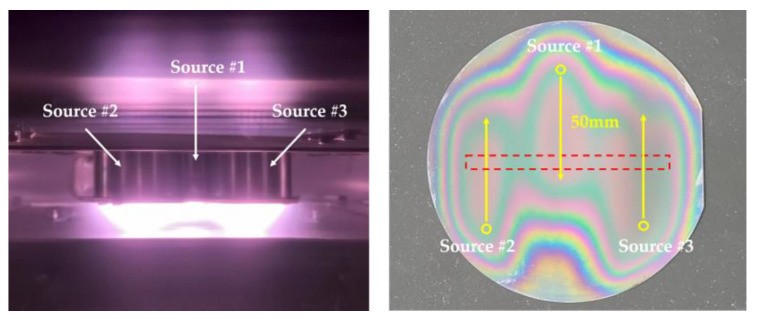
Optical images of the scanning deposition process (**left**) and the substrate after the completion of the deposition process (**right**).

To obtain a large-area diamond film, a new unit array of point plasma sources was developed, as shown in Figure 2. By adding a unit array of three point plasma sources and a reciprocating substrate, a large diamond film could be deposited. The centers of the arrayed sources were 50 mm apart, while the source and substrate were 15 mm apart. A 4-inch silicon wafer was loaded for the diamond synthesis process, and the substrate was reciprocally moved for a distance of 50 mm at 0.2 mm/s for 12 h. Diamond synthesis was performed with a CH_4_/H_2_ ratio of 0.50% (1.5 sccm CH_4_ and 300 sccm H_2_) at a microwave-applied power of 700 W, a substrate temperature of 950 °C, and a process time of 12 h. The plasma was kept stable during the deposition process. Figure 8 shows optical images of the diamond synthesis process using three single sources, as well as the substrate after the completion of the deposition process.

**Figure 9:**
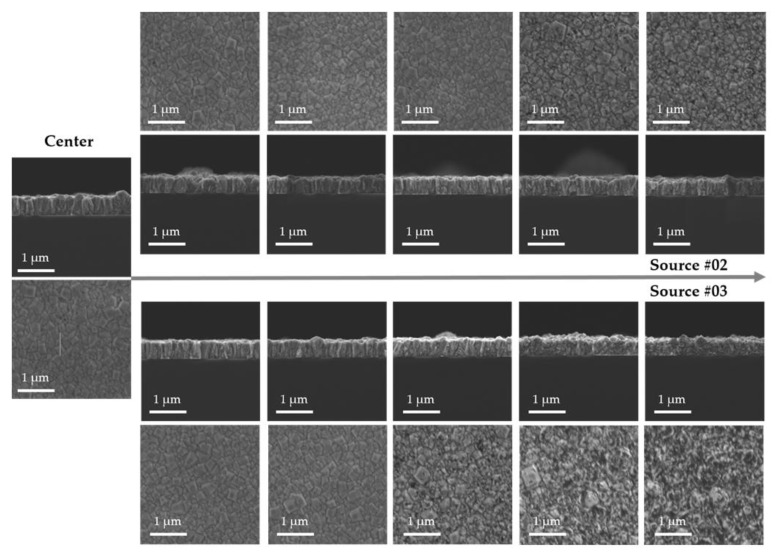

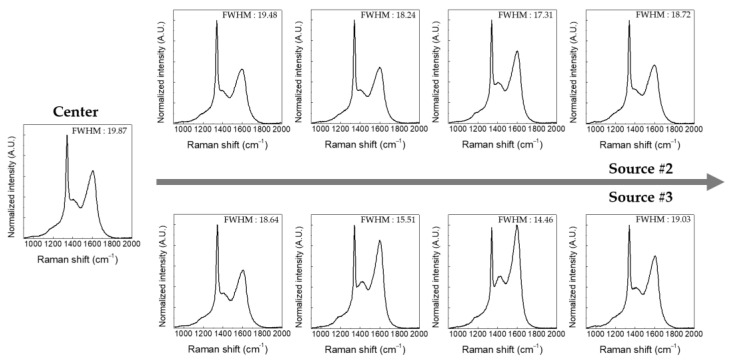
SEM images (**top**) and UV-Raman (**bottom**) spectra of sample deposited for 12 h via scanning deposition process using three point plasma sources.

After the substrate on which the thin diamond film was deposited was cut in half, the film thickness and surface morphology were measured using FE-SEM at intervals of 7 mm, as shown in Figure 9. The red dotted square indicates the actual measurement area. The FE-SEM cross-sectional measurement indicated that the thin diamond film had a thickness of 570 nm at the center, and the film thickness uniformity within 70 mm was ±6.25%.

Nanodiamonds with sizes of 100 nm were deposited with relative uniformity between sources #1 and #2. Meanwhile, the amorphous phases were deposited in a certain area between sources #1 and #3, which indicated a tendency toward nonuniformity. This could be attributed to the different mechanical and plasma characteristics of the sources or different gas distributions within the chamber. To sufficiently address this observation, additional experiments should be performed to verify the cause. The results of the sample surface analysis using UV-Raman spectroscopy are shown in Figure 9. The I_Dia_/I_G_ ratio of the UV-Raman ranged from 1.0 to 2.0, and a slightly lower diamond peak was detected at the position between sources #1 and #3, similar to the FE-SEM results. The FWHM of the diamond peak was in the range of 14–20 cm^−1^, which could be ascribed to various variables, including the crystallite size and structural disorder [32]. The decreased FWHM value could be ascribed to the smaller grain size in the region with more amorphous phases. Except for the measurement results at a specific point, the UV-Raman results were relatively similar.

## 4. Conclusions

Herein, we presented a method for diamond synthesis with theoretically infinite scalability. The characteristics of the source were observed through various experiments on the single-point plasma source. Subsequently, a method for determining the ideal plasma source arrangement was established. It was experimentally verified that a uniform diamond thin film was obtained through a reciprocating motion of the substrate and by creating a minimum unit array using three point plasma sources of 700 W each. However, the scanning process has limitations, such as a decreased deposition rate, a loss of microwave power because of the use of a coaxial cable, and decreased crystallinity of the diamond films. These limitations will be addressed in a future study.

## Figures and Tables

**Table 1:** Processing parameters for diamond synthesis.

Process Conditions	
Inlet gas	CH_4_, H_2_, Ar
Gas ratio (CH_4_/H_2_)	0.4–1.0%
Pressure	400–800 mTorr
Wafer temperature	700–950 °C
Wafer treatment	Mechanical scratch/ultrasonic (5% nanodiamond)
Microwave power	700–800 W
Plasma source	Single, triple

## Data Availability

The data presented in this study are available on request from the first or corresponding author.
